# PRE-1, a *cis *element sufficient to enhance cone- and rod- specific expression in differentiating zebrafish photoreceptors

**DOI:** 10.1186/1471-213X-11-3

**Published:** 2011-01-24

**Authors:** Maria E Morrissey, Sara Shelton, Susan E Brockerhoff, James B Hurley, Breandán N Kennedy

**Affiliations:** 1 UCD School of Biomolecular and Biomedical Sciences, UCD Conway Institute, University College Dublin, Dublin, Ireland; 2 Deptartment of Biochemistry, University of Washington, Seattle, Washington, USA

## Abstract

**Background:**

Appropriate transcriptional regulation is required for cone photoreceptor development and integrity. To date, only a few *cis*-regulatory elements that control cone photoreceptor-specific expression have been characterised. The alpha-subunit of cone transducin (TαC) is specifically expressed in cone photoreceptors and is required for colour vision. In order to better understand the molecular genetics controlling the initiation of cone photoreceptor-specific expression *in vivo*, we have utilised zebrafish to identify *cis*-regulatory elements in the upstream promoter region of the TαC gene.

**Results:**

A 0.5 kb TαC promoter fragment is sufficient to direct cone-specific expression in transgenic larvae. Within this minimal promoter, we identify photoreceptor regulatory element-1 (PRE-1), a unique 41 bp sequence. PRE-1 specifically binds nuclear factors expressed in ocular tissue. PRE-1 is not required for cone-specific expression directed from a 2.5 kb TαC promoter. However, PRE-1*-like *sequences, with potential functional redundancy, are located in this 2.5 kb promoter. PRE-1*-rho *which has the highest sequence and structural homology to PRE-1 is located in the rhodopsin promoter. Surprisingly, PRE-1 and PRE-1*-rho *are functionally distinct. We demonstrate that PRE-1, but not PRE-1*-rho*, is sufficient to enhance expression from a heterologous UV cone promoter. PRE-1 is also sufficient to enhance expression from a heterologous rhodopsin promoter without altering its rod photoreceptor specificity. Finally, mutations in consensus E-box and Otx sites prevent PRE-1 from forming complexes with eye nuclear protein and enhancing photoreceptor expression.

**Conclusions:**

PRE-1 is a novel *cis*-regulatory module that is sufficient to enhance the initiation of photoreceptor-specific gene expression in differentiating rod and cone photoreceptors.

## Background

Photoreceptors are specialised sensory neurons that enable images of the external environment to be captured. Structurally, cone and rod photoreceptors appear grossly similar. Both have outer segments rich in photosensitive membranes, inner segments rich in transport and metabolic machinery, and synaptic termini that chemically transmit light signals to downstream neurons [1]. However, cone and rod photoreceptors function distinctly. Rods are specialised to function in low-light, whereas cones enable colour vision and visual acuity [2]. These functional differences are partly explained by the distinct morphological specialisations of outer segments and synaptic termini present in rods or cones [1]. In addition, unique transcripts encode rod- or cone-specific components of the G-protein coupled receptor phototransduction pathway. This includes the α-subunit of the heterotrimeric G-protein transducin (Tα) encoded by *gnat1 *in rods and by *gnat2 *in cones. Underlining these molecular differences, these photoreceptor-specific isoforms associate with distinct forms of blindness. Mutations in the gene encoding rod transducin alpha (TαR) cause inherited night blindness whereas mutations in the gene encoding cone transducin alpha (TαC) cause achromatopsia, or total colour blindness [3,4].

During retinal neurogenesis, rod and cone photoreceptors differentiate from a population of retinal progenitor cells [5]. In 2 day post-fertilisation (dpf) zebrafish, post-mitotic cells that will differentiate into photoreceptors are visible, and transcripts for phototransduction genes can be detected [6,7]. Outer segments and synaptic ribbons, unique morphological specialisations of differentiated photoreceptors, are apparent by 2 dpf [8]. In zebrafish, visual behaviour assays indicate that functional photoreceptors are present in some larvae at 3 dpf, and that robust responses have developed in all wildtype larvae by 4-5 dpf [9,10]. Electroretinography reveals that cones are the primary functional photoreceptors at 6 dpf and that rods become functional later at 15-21 dpf [11].

Underpinning the morphological and functional development of photoreceptors is a programme of regulated gene transcription. However, relatively little is known about the *cis *transcriptional regulators that initiate cone photoreceptor-specific expression *in vivo*. In the canine retina, a 2.1 kb promoter fragment from the human red opsin gene directs reporter expression in long and medium (L/M) wavelength cones and has been applied in *proof-of-principle *gene therapy studies for inherited achromatopsia [12,13]. Although a shorter 0.5 kb fragment is inactive, the addition of 3 copies of a 35 bp red/green locus control region (LCR) is sufficient to result in weak L/M cone-specific expression *in vivo *[12]. In the murine retina, a 277-bp promoter fragment from the human TαC gene, coupled with a 214-bp IRBP enhancer, directs cone-specific expression [14]. In *Xenopus*, 0.5 kb of the red opsin promoter is sufficient to drive expression in cone photoreceptors [15,16]. Within this proximal promoter, conserved sequences required for high-level and cone-specific expression were identified, including a 13 bp ROP2 element that restricts expression to *Xenopus *cones [16]. Similarly, in heterologous analyses, a proximal 215 bp of the mouse cone arrestin promoter is sufficient to drive expression in Xenopus cone photoreceptors, whereas a shorter 147 bp cone arrestin promoter fragment directs expression in cone and rod photoreceptors [17]. Consenus TATA and CRX-binding elements are required for this photoreceptor-specific expression [17].

More recently, c*is*-regulators of cone-specific expression have been identified using transgenic approaches in the cone-abundant zebrafish [18,19]. A 4.8 kb promoter fragment of the single-copy UV opsin gene directs expression specifically to zebrafish UV cones [20,21]. A 105 bp proximal subsequence within this UV opsin promoter is sufficient to alter the specificity of a rhodopsin promoter such that it directs reporter expression in rods and UV cones [20]. In the quadruplicated array of green opsin genes, a 500 bp LCR, located ~15 kb upstream, is necessary for the activity of the four green opsin promoters *in vivo*, and is sufficient to override the cell-type specificity of a UV opsin promoter fragment by directing it to drive expression in green opsin-expressing cones [22]. *Cis*-elements repressing blue opsin expression in zebrafish UV cones and pineal cells have also been identified [23].

Using the zebrafish *gnat2 *gene encoding TαC, we are exploring the mechanisms controlling the initiation of cone photoreceptor-specific expression *in vivo*. Previously, we identified ~3.2 and ~2.5 kb promoter fragments from the zebrafish TαC gene that initiate robust EGFP expression in the four morphological subtypes of differentiating cones at 3 dpf [24,25]. Subsequently, we characterised cone photoreceptor regulatory element 1 (CPRE-1), a 20 bp enhancer element ~ 2.5 kb upstream of the TαC promoter [25]. CPRE-1 is necessary for cone-specific expression from TαC promoter fragments, but is not sufficient to enhance activity from a heterologous UV opsin promoter [25]. Here, we characterise regulatory elements in the proximal TαC promoter. We identify 0.7 and 0.5 kb promoter fragments of the TαC gene that are sufficient to initiate cone-specific expression. We then identify photoreceptor regulatory element 1 (PRE-1), a 41 bp sequence located in the 0.5 kb construct, that specifically binds eye nuclear protein. In contrast to CPRE-1, PRE-1 is not necessary for cone-specific expression from large TαC gene promoter fragments, but is sufficient to enhance the transcriptional activity of heterologous UV opsin and rhodopsin promoters in differentiating photoreceptor cells.

## Results

### Identification of Minimal Cone-Specific Promoters

**Figure 1:**
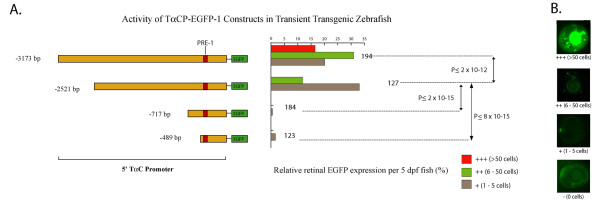
**A ~0.5 kb 5' promoter fragment of zebrafish TαC is sufficient to initiate photoreceptor specific expression in differentiating cones**. A. Schematic of the promoter-reporter portion of the zebrafish TαC promoter constructs and graphs of their corresponding activity in transgenic zebrafish larvae at 5 dpf. The number of transgenic fish scored for each construct is indicated at the right of the histograms. **B**. Examples of transient transgenic zebrafish eyes showing the activity levels used to determine the activity of the constructs.

We identify minimal zebrafish TαC/*gnat2 *promoter fragments that direct cone-specific expression in larval retinae using transient transgenic assays (Figure 1). As expected, constructs with large 3173 and 2521 bp promoter fragments drive robust EGFP expression in differentiating retinal cone photoreceptors and in the pineal [25]. However, constructs with only 717 or 489 bp of TαC promoter are sufficient to direct weak, cone-specific expression in ~1% of larvae (Figure 1a and data not shown). The absence of detectable pineal expression with the shorter constructs indicates that enhancer elements required to initiate pineal expression are located between -2521 and -717 bp.

### PRE-1 Specifically Binds Eye Nuclear Factors

**Figure 2:**
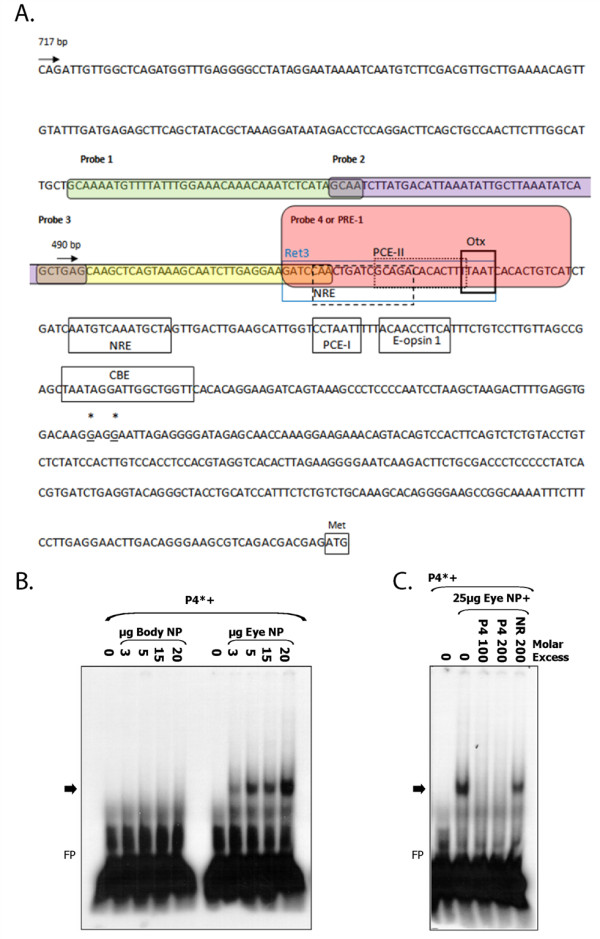
**Identification of PRE-1 in TαCP. A**. Annotated zebrafish TαC proximal promoter sequence. The sequence of probes 1-4 used in electrophoretic mobility shift assays are highlighted by rounded boxes. The PRE-1 (probe 4) sequence is highlighted in pink. The most 5'-end of zebrafish TαC ESTs (accession numbers DY550630, BG307391) are indicated by asterisks and underlined. The translation start codon is indicated by a box and methionine (Met). Putative trans-factor binding sites for Ret 3, NRE, PCE-1, PCE-II, Otx, CBE, Nr2e3 and E-boxes, based on homology to known *cis*-elements are indicated by boxes [26-29,33,36,51,52]. *NRE, Nrl Response Element. PCE, photoreceptor conserved element. CBE, Crx-binding element*. **B**. PRE-1 specifically binds nuclear factors expressed in ocular tissue. Electrophoretic mobility shift assay of radiolabelled PRE-1 (TαC probe 4) incubated with increasing concentrations of zebrafish eye or body nuclear protein. PRE-1 binds nuclear protein isolated from adult zebrafish eyes but not body nuclear protein. **C**. Competition analyses indicate that binding of trans-factor(s) is specific because unlabelled PRE-1 at 100 or 200-fold molar excess is able to compete away binding of eye nuclear protein to labelled PRE-1, whereas an unlabelled non-related competitor at 200-fold molar excess is not. *Nuclear protein (NP), non-related (NR), probe 4 (P4 or PRE-1), free probe (FP)*. Asterisks indicate radiolabelled probe and arrow indicates the DNA-protein complex of interest.

To identify *cis*-elements in the proximal promoter region that bind nuclear factors, we performed electrophoretic mobility shift assays (Figure 2). Four probes, corresponding to sequences within the proximal 0.7 kb of the zebrafish TαC promoter, were incubated with nuclear extracts isolated from adult zebrafish eyes (Figure 2). No specific complexes were detected with probes 1-3 (data not shown). However, probe 4 forms a complex with nuclear factors contained in zebrafish ocular tissue (Figure 2B). When probe 4 is incubated with increasing concentrations of eye nuclear extract, there is a dose-dependent increase in the intensity of the shifted complex. In contrast, probe 4 does not form a complex with nuclear extract isolated from mid-body tissue. The specificity of the interaction between probe 4 and eye nuclear factors was confirmed in competition assays. Whereas complex formation between radio-labelled probe 4 and eye nuclear factors was completely abolished by competition with 100 or 200 M excess of unlabeled probe 4, complex formation was not inhibited with 200 M excess of a non-related competitor probe (Figure 2C). These results suggest that the observed probe 4 complexes are tissue- and sequence-specific. Interestingly, probe 4, which was subsequently named photoreceptor regulatory element 1 *(*PRE-1), is located ~150 bp upstream of the predicted zebrafish TαC transcription start site, and adjacent to the 5' border of the minimal 489 bp construct that drives cone-specific expression.

### Multiple PRE-1 Sequences are Required for Complex Formation with Eye Nuclear Factors

**Figure 3:**
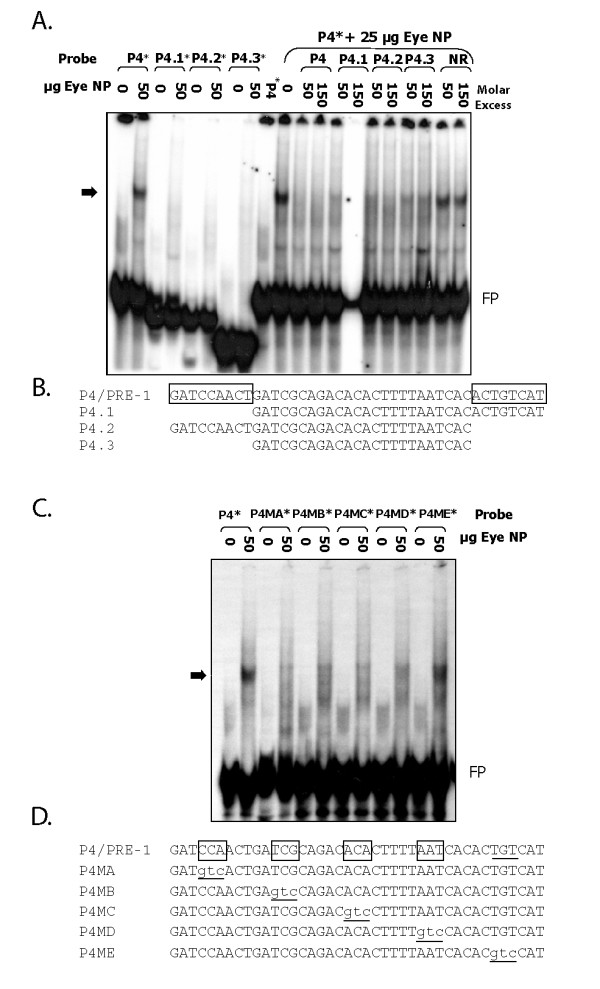
**The majority of the 41 bp PRE-1 sequence is required for binding of nuclear factors *in vitro***. Large deletions and 3 bp mutations of PRE-1 eliminate trans-factor(s) binding. **A**. Electrophoretic mobility shift assay demonstrates that PRE-1 deletion probes P4.1-3 are unable to form a complex with eye nuclear protein but are sufficient to compete away factors from binding to full length P4 at the indicated molar excess of unlabelled probe. **C**. Electrophoretic mobility shift assay demonstrates that mutant PRE-1 probes P4MA-D are unable to bind eye nuclear protein but P4ME is, indicating that 3 bp sequences mutated in A-D are necessary for trans-factor(s) binding to PRE-1. **B, D**. Sequence of PRE-1 deletion and mutant probes. Nucleotides required for binding are indicated by boxes and 3 bp mutated sequences are in lowercase and underlined*. Nuclear protein (NP), non-related (NR), probe 4 (P4 or PRE-1), free probe (FP)*. Asterisks indicate radiolabelled probe and arrow indicates the DNA-protein complex of interest.

Bioinformatic analyses reveal that probe 4/PRE-1 has sequence homology to known or predicted *cis*-regulators of photoreceptor expression (Figure 2 and Additional file 1, Figure S1), including an E-box (6 of 6 bp), an Otx element (6 of 6 bp), Ret 3 (18 of 28 bp), NRE (10 of 12 bp), PCE-II (9 of 12 bp), rhodopsin enhancer element (24 of 40 bp) and the 5' end of the zebrafish green opsin LCR (29 of 46 bp) [22,26-30]. To refine the PRE-1 sequences required for complex formation with eye nuclear protein, and to evaluate the significance of the homologous *cis*-sequences, probes containing deletions or mutations were tested in electrophoretic mobility shift assays (Figure 3). PRE-1 probes with a 9 bp deletion at the 5' end, a 7 bp deletion at the 3' end or both deletions, fail to form complexes with eye nuclear protein (Figure 3A, B). However, when added as 50 or 150 M excess cold competitor, the three deletion probes prevent eye nuclear protein forming complexes with radio-labelled wildtype PRE-1 (Figure 3A). This competitive inhibition is specific, as 50 or 150 M excess of a non-related probe does not prevent complex formation (Figure 3A). Thus, 7 and 9 bp terminal sequences are essential for ocular trans-factors to form a DNA-protein complex with PRE-1 (Figure 3B). Five mutant probes with 3 bp mutations distributed throughout PRE-1 were also analysed (Figure 3C, D). Four of the mutant probes (P4MA-D) fail to form complexes with eye nuclear protein. In contrast, mutant probe P4ME forms an equivalent complex as the wildtype PRE-1 probe. In summary, the mutant analyses identify four distinct 3 bp sequences, distributed across the majority of PRE-1, that are required for complex formation with eye nuclear factors (Figure 3D).

### PRE-1 is Not Required for Activity from a 2.5 kb TαC promoter construct

**Figure 4:**
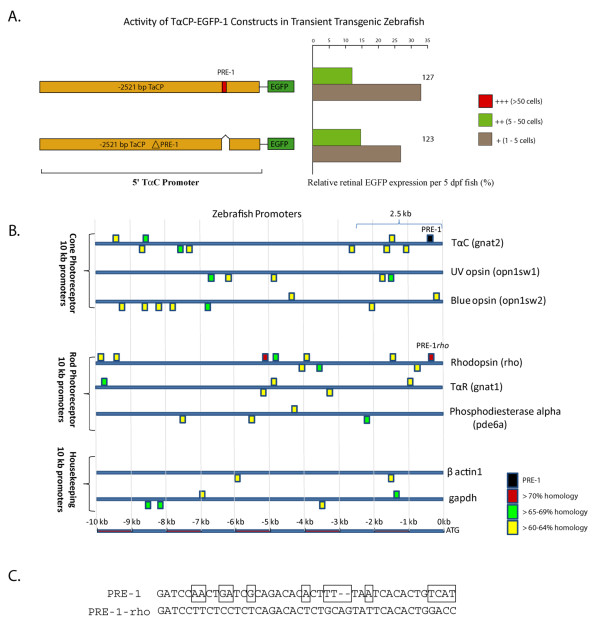
**PRE-1 is not required for ~2.5 kb TαC promoter activity *in vivo***. **A**. Schematic of the 2521 bp TαC wildtype and PRE-1 deletion constructs and graph of their activity in transient transgenics at 5 dpf. Deletion of PRE-1 has no significant effect on the activity levels of the fragment. The number of transgenic fish scored for each construct is indicated at the right of the histograms. A red box highlights the PRE-1 element. **B**. Schematic showing the location of PRE-1-*like *sequences in 10 kb promoter fragments of zebrafish photoreceptor and housekeeping genes with common gene name to the right and gene symbol in brackets. Bioinformatic alignments reveal several PRE-1-*like *sequences in the ~2.5 kb TαC promoter suggesting redundancy. PRE-1-*like *sequences are also located in promoter fragments of housekeeping genes. However, the highest sequence similarity was found in the proximal rhodopsin promoter. **C**. Clustal W pairwise alignment of PRE-1 and PRE-1-*rho *with mismatched sequences highlighted in boxes.

To determine if PRE-1 is required for initiation of transgene expression in cone photoreceptors, we tested the *in vivo *activity of a reporter construct containing an internal PRE-1 deletion (Figure 4A). In comparison to the wildtype 2521-bp TαC promoter construct, no significant differences in the pattern of expression is observed when PRE-1 is deleted. Both constructs are expressed in 42-44% of injected embryos and there is no significant difference in the number of EGFP-expressing cells observed in the retina with these constructs. This may reflect functional redundancy with additional PRE-1-like sequences. Indeed, bioinformatic alignments to the 2521-bp TαC promoter reveals 3 sequences with >60% identity to PRE-1 (Figure 4B). Additional searches of zebrafish genes with enriched expression in zebrafish rod or cone photoreceptors reveals the presence of multiple sequences in the upstream promoter region with >60% identity to PRE-1 (Figure 4B). However, whether these PRE-1*-like *sequences have unique roles in photoreceptor gene regulation is questionable as sequences with >60% identity to PRE-1 are also found in the upstream promoter regions of the *β-actin *and *gapdh *encoding housekeeping genes (Figure 4B). In addition, although one PRE-1*-like *sequence is typically found within 10 kb promoter regions of genes encoding TαC orthologues, there is no evidence of evolutionary pressure to retain a homologous PRE-1 sequence in a conserved location (Additional file 2, Figure S2). By selecting PRE-1*-like *sequences whose identity score is greater than those in the housekeeping genes, we discovered that the zebrafish rhodopsin gene has distal and proximal PRE-1*-like *sequence with 71% and 72% identity, respectively (Figure 4B, C). Notably, compared to PRE-1, the proximal rhodopsin PRE-1 is present at an equivalent spatial location and orientation relative to the gene transcription initiation site, and is designated PRE-1*-rho*_._

### PRE-1 Is Sufficient To Enhance Photoreceptor-Specific Promoters

**Figure 5:**
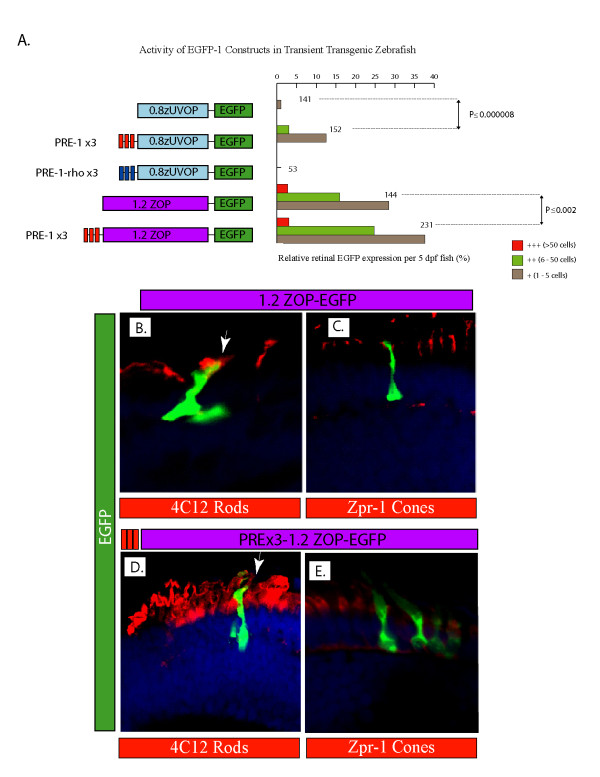
**PRE-1 is sufficient to enhance reporter expression from photoreceptor-specific promoters**. **A**. Schematic of the promoter-reporter regions of chimeric constructs. Three copies of PRE-1 (red boxes) are sufficient to enhance expression from both cone and rod promoter fragments, whereas three copies of PRE-1-*rho *(navy boxes) are unable to enhance expression from the cone promoter fragment. Blue and purple rectangles represent zebrafish UV opsin and rhodopsin promoter fragments respectively, upstream of EGFP. **B-E**. Confocal micrographs of retinal sections from 5 dpf embryos injected with 1.2 kb ZOP-EGFP or (3x PRE-1)-1.2 kb ZOP-EGFP constructs and stained with 4C12 and zpr-1 antibodies. EGFP-positive cells co localize (arrows) with rod photoreceptors (4C12 antibody), but not with cone photoreceptors (zpr-1 antibody). Nuclei are counter-stained with DAPI.

To circumvent potential functional redundancy issues in the deletion constructs, the ability of multiple PRE-1 and PRE-1*-rho *modules to enhance the *in vivo *activity of heterologous promoters was tested instead (Figure 5). Chimeric constructs comprising of 3 copies of PRE-1 or PRE-1*-rho *upstream of the previously described zebrafish UV opsin or rhodopsin promoters were tested in transient transgenesis assays [25,31]. A 0.8 kb zebrafish UV opsin promoter fragment directs weak, but tissue-specific expression in differentiating UV cone photoreceptors (Figure 5A). PRE-1 functionality is unmasked in this assay, as three copies of PRE-1 are sufficient to significantly enhance the reporter activity from the UV opsin promoter (Figure 5A). Surprisingly, a chimera with three copies of PRE-1*-rho*, which has high sequence identity to PRE-1, is unable to enhance activity of the UV-promoter (Figure 5A). PRE-1 is also able to enhance the *in vivo *activity of a rod photoreceptor-specific promoter. A 1.2 kb zebrafish rhodopsin promoter fragment drives moderate reporter expression, specifically in differentiating rod photoreceptors (Figure 5A). In chimeric constructs, three copies of PRE-1 are also sufficient to significantly enhance reporter expression from this rod-specific promoter (Figure 5A). Analysis of retinal sections from zebrafish injected with the rod chimeric construct indicates that PRE-1 increases the number of rod photoreceptor cells expressing the EGFP reporter, without altering rod photoreceptor-specificity (Table 1). The 1.2 kb zebrafish rhodopsin promoter fragment on its own (Figure 5B, C) or with three copies of PRE-1 upstream (Figure 5D, E) drives expression in EGFP-positive cells that co-localise with 4C12, a marker for rod photoreceptors (Figure 5B, D), but not with zpr-1, a marker for cone photoreceptors (Figure 5C, E).

**Table 1:** PRE-1 is Not Sufficient to Alter the Specificity of a Rod-Specific Promoter

	Colocalization	Colocalization
**Construct Injected**:	**4C12 (rods)**	**Zpr-1 (cones)**

1.2kb-ZOP-EGFP	**10 **(12)	**0 **(5)

(3x PRE-1)(1.2kb-ZOP)-EGFP	**9 **(9)	**0 **(7)

Parentheses indicate the number of EGFP-positive cells and the number that also labelled with cone or rod photoreceptor antibodies is highlighted in bold.

## Discussion

In order to better understand the molecular genetics initiating cone photoreceptor-specific expression *in vivo*, we have utilised the zebrafish model to identify *cis*-regulatory elements in the upstream promoter region of the TαC gene. During phototransduction, TαC is required for cone opsins to activate phosphodiesterase. Mutations in the human TαC gene cause total colour blindness, and mutations in the zebrafish orthologue also result in cone-based blindness [4,32].

**Figure 6:**
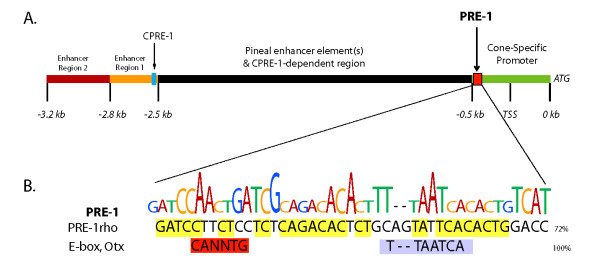
**Transcriptional Regulation of Zebrafish TαC**. **A**. Schematic showing the location of PRE-1 and other upstream promoter regions known to regulate zebrafish TαC transcription. A green bar indicates the minimal cone-specific promoter and PRE-1 is represented by a red square. Upstream enhancer regions are indicated by red and orange rectangles, and CPRE-1 by a blue box. The region containing pineal enhancer(s) and CPRE-1 -dependent regions are indicated by a black rectangle. ATG is the translation start site and TSS is the predicted transcription start site. **B**. Schematic of the DNA bases in PRE-1 critical for complex formation with nuclear protein or for enhancement of reporter activity. The size of each letter is proportional to the importance of the nucleotide in *in vitro *EMSA and *in vivo *reporter assays. The alignment of critical PRE-1 residues to consensus E-box and Otx sites and to PRE-1-*rho *is also shown.

The zebrafish TαC gene is specifically expressed in all cone photoreceptor types by 2.5 dpf and therefore, contains the *cis*-regulatory elements to direct transgene expression in all differentiating and mature cones. Previously, we demonstrated that a 3.2 kb promoter fragment of the TαC gene initiates robust transgene expression in differentiating cones and identified CPRE-1, a 20 bp distal enhancer [24,25]. Here, we characterise the proximal promoter of the zebrafish TαC gene and demonstrate that a 0.5 kb fragment is sufficient to initiate cone-specific expression. In this proximal 0.5 kb promoter fragment, we identify a photoreceptor regulatory element, PRE-1. This 41 bp sequence is specifically bound by nuclear factors expressed in ocular tissue. PRE-1 is sufficient to enhance photoreceptor-specific expression from heterologous UV cone opsin and rhodopsin promoters. Typical of classical enhancers, PRE-1 is spatially independent, enhancing expression when upstream of a 0.8 kb UV opsin or a 1.2 kb rhodopsin promoter. The combined activity of two distal enhancers, an uncharacterised enhancer dependent region at -~2.5/0.8 kb and the proximal PRE-1 characterised here, likely contribute significantly to the robust tissue-specific expression of TαC in cones (Figure 6) [25].

In comparison to *cis*-regulators of photoreceptor expression previously described in zebrafish, PRE-1 has unique characteristics. Cone photoreceptor regulatory element 1 (CPRE-1), a short distal enhancer of TαC, is necessary for high levels of promoter activity in cones, but is not sufficient to enhance expression from heterologous promoters [25]. A 500 bp distal LCR in the green opsin array or a 105 bp sequence in the proximal UV opsin promoter are sufficient to enhance expression levels and to override specificity of heterologous promoters [20,22]. In contrast to these, PRE-1 is sufficient to enhance the transcriptional activity of heterologous promoters, but not to override rod photoreceptor specificity. Despite these functional differences, a unifying feature of the RH2-LCR, the UV opsin enhancer, a blue opsin regulatory element and PRE-1, is the presence of consensus sites for Otx [20,22,23]. Overall, this indicates that PRE-1 binding factors, which enhance basal promoter activity, are expressed in both rod and cone photoreceptors, but that additional *cis*-elements dictate tissue specificity.

Although PRE-1 is sufficient to enhance photoreceptor-specific expression, PRE-1 is not required to direct cone-specific expression from a 2.521 kb TαC promoter fragment. The difference is likely to result from functionally redundant *cis*-elements. Indeed, three sequences homologous to PRE-1 are present in the 2.521 kb promoter fragment. However, caution is necessary when associating sequence homology with functional equivalency. This is best exemplified by PRE-1*-rho *which has the highest sequence identity to PRE-1, but which is unable to enhance activity from a heterologous promoter, and thus is functionally distinct.

PRE-1 has consensus sites for known *cis*-regulators of photoreceptor expression including E-box, Otx, NRE, PCE-II and Ret 3 elements. E-box motifs, bound by basic helix-loop-helix transcription factors, have been reported in the promoter regions of several photoreceptor expressed genes and PCE-II is sufficient to drive photoreceptor-specific expression in *Xenopus *[29,33,34]. Ret 3 is a DNaseI protected site in the distal rhodopsin enhancer region (RER) and forms a complex with factors expressed specifically in retinal tissue [27]. The transcription factor, neural retina-specific leucine zipper (Nrl), is expressed in rods, and the presence of an Nrl response element (NRE) in this enhancer of rod and cone photoreceptor expression is a little unexpected. In conjunction with the cone-rod homeobox (Crx) transcription factor, Nrl promotes rod opsin expression in rods, and by regulating expression of the nuclear receptor Nr2e3, Nrl suppresses the expression of cone genes in rods [28,35]. This suggests that a factor other than Nrl binds PRE-1, that the activity of Nrl alters depending on the presence of adjacent *cis*-elements, or that the consensus NRE in PRE-1 is non-functional. In contrast, the presence of an Otx element, a binding site for the CRX transcription factor is unsurprising, as Crx regulates expression of rod and cone genes in mice, zebrafish and *Xenopus *[26,36-38]. PRE-1 also shows homology with the LCR regulating expression of the zebrafish green opsin genes and the bovine rhodopsin enhancer region [22,30]. Of all these putative regulatory sequences, functional analyses indicate that the consensus Otx and E-box sites are most important (Figure 6).

It is likely that PRE-1 is bound by Crx at the Otx site and a basic helix-loop-helix factor at the E-box site. This is supported by the requirement of consensus Otx and E-box sites for complex formation of ocular nuclear factors with PRE-1. Furthermore, the consensus Otx and E-box sites are mutated in PRE-1-*rho *which shows high sequence conservation, but no functional conservation, with the PRE-1 identified in the TαC promoter (Figure 6). Crx is expressed in rod and cone photoreceptors and regulates gene expression in conjunction with co-expressed transcription factors including Nrl, Nr2e3, Sp4, Sp1 and Qrx [39-41]. Crx is known to bind in the vicinity of the mouse TαC gene [42]. In addition, reducing or eliminating Crx expression in zebrafish morphants and mouse knockouts, results in a significant down-regulation of TαC expression [36,43]. Crx also recruits histone acetyltransferases [44]. However, it is unlikely that PRE-1 functions via histone modifications in our transient assays where the majority of the episomal DNA is predicted not to be bound by chromatin [45]. A basic helix-loop-helix factor that potentially binds PRE-1 is Zash1a, an achaete-scute complex-like 1 (Ascl1) orthologue. This speculation is supported by the known binding of the Mash-1 orthologue to an E-box in the rodent rhodopsin promoter, the ability of Mash-1 to induce retinal progenitors to a photoreceptor cell fate and the robust expression of zash1a in the developing zebrafish eye [33,46,47].

## Conclusions

Deciphering the genomic DNA sequences that control tissue-specific gene expression provides fundamental insights into the molecular mechanisms enabling complex systems to develop and function. Downstream translations include engineering of *cis*-sequences to appropriately control gene expression for emerging gene therapies. In this study, we identify and characterise photoreceptor regulatory element-1 (PRE-1), a proximal 41 bp sequence, sufficient to enhance photoreceptor-specific gene expression in differentiating rods and cones. Future studies will aim to identify the *cis*-sequences and trans-factors that co-operate with PRE-1 to regulate cone-specific expression.

## Methods

### Animals

All studies using animals were approved by the UCD Animal Research Ethics Committee (AREC-P-07-75).

### Generation of Reporter Constructs

The plasmid templates for all constructs were previously published; -1.2-kbp ZOP-EGFP-1 [31], -2521-bp zTαCP-EGFP-1 and -897-bp zUVOP-EGFP-1 [25]. The internal mutant deleting PRE-1 was constructed by introducing PCR fragments into -2521-bp zTαCP-EGFP-1 digested with *BamHI *and *NdeI*. For the chimeric constructs, 3 copies of PRE-1 and PRE-1*-rho *were synthesised (MWG) and subcloned immediately upstream of the -897-bp UV opsin minimal promoter digested with *EcoRI *and *PstI *or -1.2 kb rhodopsin promoter digested with *XhoI *and *EcoRI*. All constructs were confirmed by DNA sequencing.

### Generation of Transient Transgenic Zebrafish

To generate transient transgenics, ~7.5 pg of covalently closed circular plasmid was injected into zebrafish embryos at the 1- to 2-cell development stage. These reporter constructs were made up to a final concentration of 25 ng/μl labelled with 0.1% phenol red in 0.1 M Tris (pH 7.9) as tracer dye for injection into embryos positioned on agarose chambers using a pneumatic Pico-injector system [48]. Borosilicate microcapillaries (1.0/0.58 outer/inner diameter) were pulled using a vertical needle puller (Narishige PC-10). Embryos were reared until 5 dpf in water containing 0.003% 1-phenyl-2-thiourea that was changed daily to inhibit melanin production [48].

### Quantification of EGFP Expression Directed by Reporter Constructs

Fish at 5 dpf were anesthetized with tricaine (MS-222), placed in a depression slide and analysed to score construct activity using a Zeiss Axioplan 2 fluorescence microscope under a 10X objective. True EGFP signal and autofluorescence was distinguished by comparing the FITC and rhodamine filter sets. One of three ordinal activity levels was assigned; >50 EGFP-positive cells in the eye assigned "+++"; 5-50 EGFP-positive cells assigned "++"; 1-5 EGFP-positive cells assigned "+" and no EGFP-positive cells assigned an activity level of "-" [20,25]. Statistical analysis was performed using a two-tailed Student's t-test and p-values ≤0.05 considered significant.

### Retinal Section Immunolabelling

Fish screened for EGFP expression were fixed overnight in 4% paraformaldehyde at 4°C. For cryoprotection, samples were washed in increasing concentration of sucrose in 0.1 M phosphate buffer (up to 20%) and placed in 20% sucrose overnight at 4°C. For embedding, samples were incubated for 30 minutes with increasing ratios (1:2, 1:1, 2:1) of OCT embedding medium (Tissue-Tek) to 20% sucrose and finally snap-frozen in 100% Tissue-Tek OCT [49]. 12-μm sections cut on a HM 550 cryostat were mounted onto Superfrost slides and dried at room temperature. Sections were rehydrated in 0.1 M phosphate buffer (pH 7.4), placed for one hour in blocking buffer (5% goat serum, 1% bovine serum albumin, 1% Triton X-100 in 0.1 M phosphate buffer) and incubated overnight with primary antibodies in blocking buffer at 4°C. Primary antibodies used were zpr-1 (dilution 1:200, Oregon Monoclonal Bank) and 4C12 (dilution 1:200, courtesy of JM Fadool). Sections were washed three times for 5 minutes with 0.1% Tween in 0.1 M phosphate buffer and were incubated for one hour with secondary antibodies in blocking buffer. Sections were rinsed three times in 0.1 M phosphate buffer, incubated for 5 minutes with 300 nM DAPI, rinsed again and finally mounted in Aqua-Poly/Mount medium. Sections were analysed by an LSM510 Zeiss confocal microscope.

### Electrophoretic Mobility Shift Assay

Nuclear protein extracts were prepared from adult zebrafish eyes and mid body in the presence of protease inhibitors as previously described [25]. Probes were generated by annealing complementary oligonucleotides designed with 5' overhangs. Probes were radiolabelled with ^32^-P using Klenow Exo- (Strategene) and purified on Sephadex G-50 columns. For gel shift analysis, nuclear protein was incubated on ice with 2 μg of non-specific competitor poly[dI-dC] and appropriate unlabelled competitor for 10 minutes at 4°C prior to incubation with the labelled probe for 30 minutes with increasing concentrations of nuclear protein. For competition assays, cold competitor was added prior to the addition of labelled probe in 50, 100, 150 or 200 fold molar excess. The reactions were electrophoresed on 4-8% non-denaturing polacrylamide gels followed by autoradiography.

### DNA Sequence Analysis

10 kb 5' promoter sequences of zebrafish photoreceptor phototransduction genes (*gnat2 *ENSDARG00000042529*, opn1sw1 *ENSDARG00000045677*, opn1sw2 *ENSDARG00000017274*, rho *ENSDARG00000002193*, gnat1 *ENSDARG00000044199 *and pde6a *ENSDARG00000075911) and housekeeping genes (*Bactin1 *ENSDARG00000037746 *and gapdh *ENSDARG00000043457), and *gnat2 *orthologues (human ENSG00000134183, gorilla ENSGGOG00000013316, bovine ENSBTAG00000013017, rat ENSRNOG00000019296, mouse ENSMUSG00000009108, cat ENSFCAG00000015596, dog ENSCAFG00000019823 and fugu ENSTRUG00000005471) were obtained from the ENSEMBL database. These 10 kb sequences were aligned with the 41 bp PRE-1 sequence in both orientations using Clustal W and of the top 20 alignments identified by Clustal W [50], only sequences with a pairwise alignment score greater than 0.60 were considered significant. Previously reported photoreceptor elements were also aligned with the PRE-1 sequence using Clustal W and those with a pairwise alignment score greater than 0.60 were considered significant.

## List of abbreviations used

CBE: Crx-binding element; CPRE-1: cone photoreceptor regulatory element 2; dpf: days post fertilization; EGFP: enhanced green fluorescent protein; IRBP: interphotoreceptor retinoid binding protein; LCR: locus control region; NRE: Nrl response element; PRE-1: photoreceptor regulatory element 1; PCE: photoreceptor conserved element; TBS: Tris-buffered saline; TαC: cone transducin α subunit; TαR: rod transducin α subunit; ZOP: zebrafish rod opsin promoter; zUVOP: zebrafish UV opsin promoter

## Authors' contributions

MEM drafted the manuscript and tested the *in vivo *activity levels of constructs. BNK and SS performed the electrophoretic mobility shift assays. BNK, SB and JH conceived, designed and supervised the study, and contributed to writing the manuscript. All authors read and approved the final manuscript.

## Supplementary Material

Additional file 1 **Figure S1 - Alignment of PRE-1 Sequence to Known Photoreceptor Regulatory Elements**. Schematic showing regions of homology of PRE-1 to known photoreceptor *cis*-elements. Click here for file

Additional file 2 **Figure S2 **- **Location of PRE-1-Like Sequences in GNAT2 Orthologues**. Schematic showing the location of PRE-1-*like *sequences in 10 kb promoter fragments of *gnat2 *orthologues from human, gorilla, cow, rat, mouse, cat, dog and fugu. Click here for file
