# Supplementary figures and images for: PRE-1, a cis element sufficient to enhance cone- and rod- specific expression in differentiating zebrafish photoreceptors
Source: BMC Dev Biol. 2011 Jan 24;11:3. doi: 10.1186/1471-213X-11-3 (PMC3036647; doi:10.1186/1471-213X-11-3)

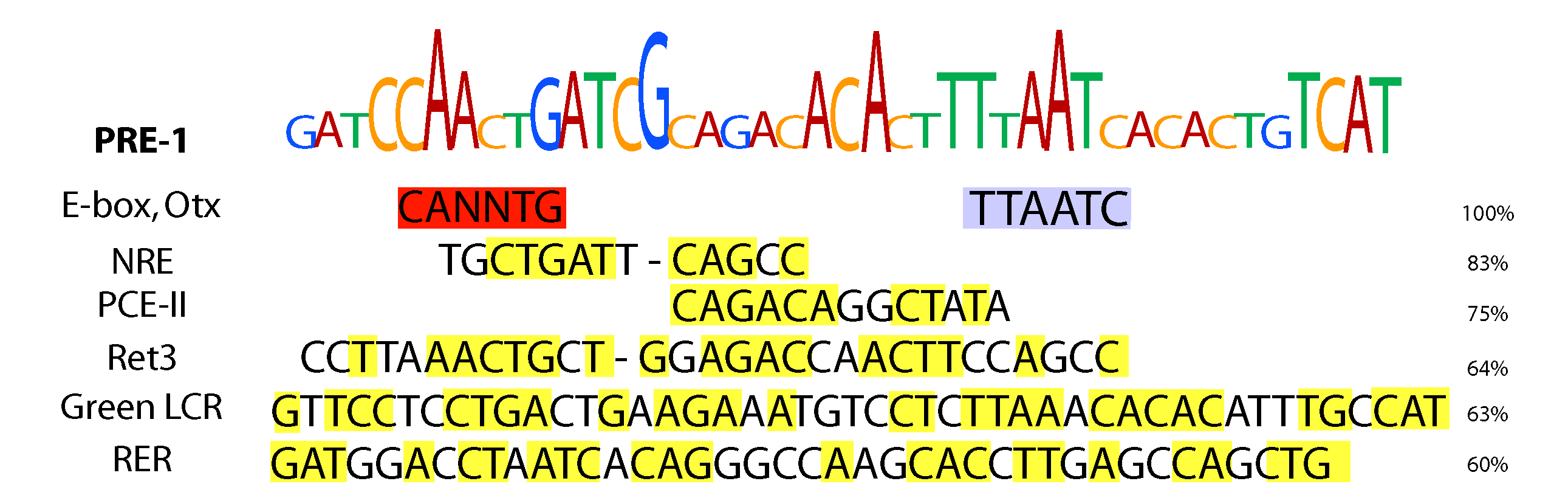

Supplement: Additional file 1 — Figure S1 - Alignment of PRE-1 Sequence to Known Photoreceptor Regulatory Elements. Schematic showing regions of homology of PRE-1 to known photoreceptor cis-elements. [file 1471-213X-11-3-S1.TIFF]

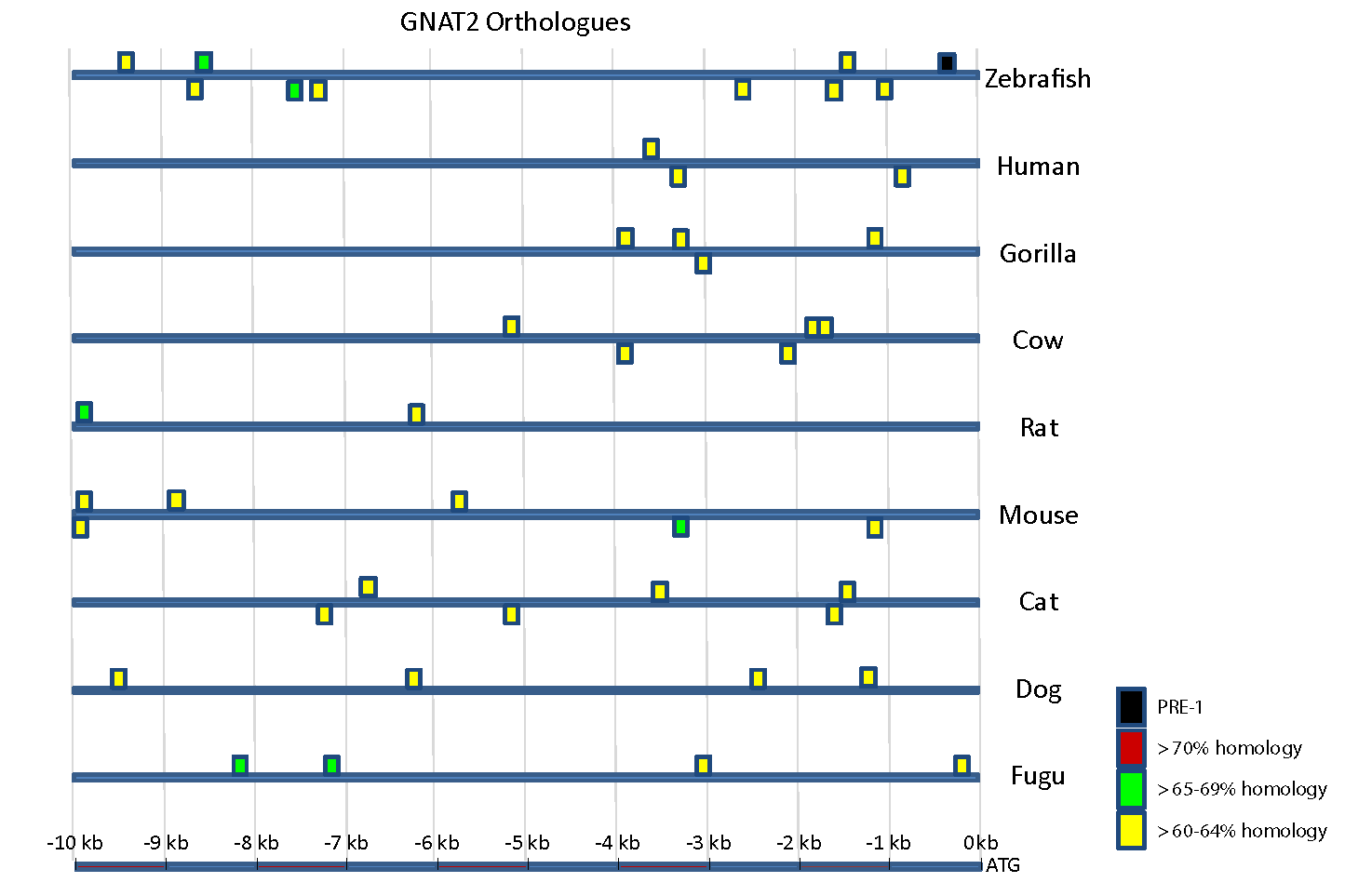

Supplement: Additional file 2 — Figure S2 - Location of PRE-1-Like Sequences in GNAT2 Orthologues. Schematic showing the location of PRE-1-like sequences in 10 kb promoter fragments of gnat2 orthologues from human, gorilla, cow, rat, mouse, cat, dog and fugu. [file 1471-213X-11-3-S2.TIFF]
